# Advancements in Detection and Mitigation Strategies for Petroleum-Derived Contaminants in Aquatic Environments: A Comprehensive Review

**DOI:** 10.3390/s24113284

**Published:** 2024-05-21

**Authors:** Hugo Duarte, María José Aliaño-González, Anabela Romano, Bruno Medronho

**Affiliations:** 1 MED—Mediterranean Institute for Agriculture, Environment and Development, CHANGE—Global Change and Sustainability Institute, Faculdade de Ciências e Tecnologia, Universidade do Algarve, Campus de Gambelas, Ed. 8, 8005-139 Faro, Portugal; hmduarte@ualg.pt (H.D.); aromano@ualg.pt (A.R.); bfmedronho@ualg.pt (B.M.); 2 Departamento de Química Analítica, Facultad de Ciencias, Universidad de Cádiz, 11510 Cadiz, Spain

**Keywords:** petroleum-derived products, contamination, water, sensors, chromatography, spectroscopy, chemometrics, foams

## Abstract

The exponential increase in the production and transportation of petroleum-derived products observed in recent years has been driven by the escalating demand for energy, textiles, plastic-based materials, and other goods derived from petroleum. Consequently, there has been a corresponding rise in spills of these petroleum derivatives, particularly in water sources utilized for transportation or, occasionally, illegally utilized for tank cleaning or industrial equipment maintenance. Numerous researchers have proposed highly effective techniques for detecting these products, aiming to facilitate their cleanup or containment and thereby minimize environmental pollution. However, many of these techniques rely on the identification of individual compounds, which presents significant drawbacks, including complexity of handling, subjectivity, lengthy analysis times, infeasibility for in situ analysis, and high costs. In response, there has been a notable surge in the utilization of sensors or generalized profiling techniques serving as sensors to generate characteristic fingerprints of these products, thereby circumventing the aforementioned disadvantages. This review comprehensively examines the evolution of techniques employed for detecting petroleum-derived products in water samples, along with their associated advantages and disadvantages. Furthermore, the review examines current perspectives on methods for the removal and/or containment of these products from water sources, to minimize their environmental impact and the associated health repercussions on living organisms and ecosystems.

## 1. Introduction

The frequency and magnitude of marine petroleum-derived product (PDP) spills have increased dramatically in recent years due to the combined effects of escalating population growth and industrial expansion [1,2]. The production, transportation, and consumption of petroleum-based products have increased in tandem with the rising global energy demand, which has amplified the likelihood of accidents and incidents along the supply chain [3]. Furthermore, the phenomenon of rapid urbanization and industrialization has led to the proliferation of infrastructure, such as pipelines, refineries, and marine terminals, which has augmented the probability of leaks, ruptures, and operational failures [4,5]. Furthermore, the intensification of drilling and exploration activities in sensitive marine ecosystems has increased the risk of catastrophic spills with far-reaching ecological and economic ramifications [6].

The spillage of PDPs into water bodies represents a significant environmental challenge with serious potential ecological and socio-economic consequences [7]. As previously stated, such spills can occur due to a variety of causes, including accidents during transport and extraction, deliberate acts of vandalism, and/or industrial negligence to avoid cleanup fees [8]. The consequences of these incidents are manifold and immediate, affecting aquatic ecosystems, human health, and the livelihoods of communities dependent on marine resources.

The spillage of PDPs can result in a multitude of environmental impacts, including the permanent alteration of physical, chemical, and biological processes [9]. The composition of crude petroleum and its derivatives is complex, containing a diverse array of hydrocarbons, heavy metals, and other toxic compounds. These substances can persist in the environment for extended periods, disrupting natural processes and endangering the health of aquatic organisms [10,11]. Furthermore, these products can form a film on the water’s surface, which obstructs the passage of light, thus affecting the marine environment in different ways. Moreover, the release of volatile organic compounds (VOCs) into the atmosphere worsens the environmental impact of these incidents, contributing to air pollution and atmospheric deposition.

The identification and containment of PDP spills present significant challenges for environmental scientists and regulatory authorities. The magnitude and unpredictability of these events often make rapid response difficult. Contaminants can spread quickly through surface waters and penetrate sediments and shoreline habitats [12]. The detection and monitoring of PDPs are also compromised by the typical diversity of hydrocarbons present, requiring sophisticated analytical techniques and interdisciplinary collaboration [13]. Similarly, conventional strategies for PDP containment, such as booms and skimmers, face relevant limitations and are often not suitable for mitigating the spread of petroleum contaminants in open water. The use of chemical dispersants and sorbents, although intended to expedite biodegradation and removal of PDPs from affected areas [14], may introduce further ecological risks and uncertainties.

In light of these challenges, there is an urgent need for innovative approaches capable of addressing the inherent complexities of PDP spills in water. This requires an interdisciplinary research effort that integrates knowledge from fields such as environmental science, engineering, chemistry, and statistical analysis.

**Figure 1:**
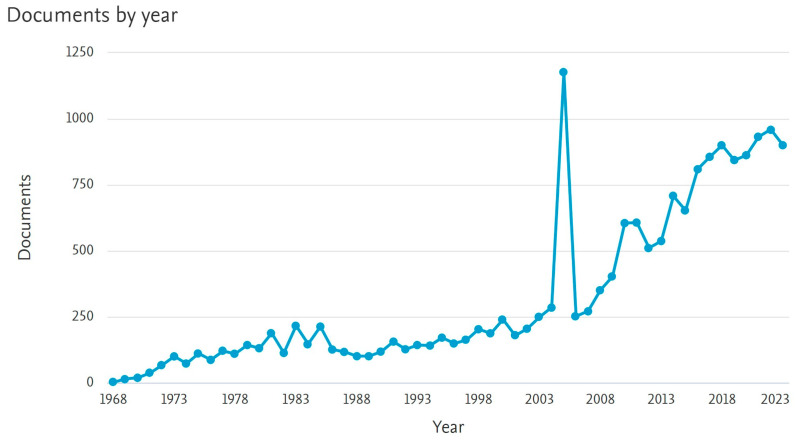
The number of documents published related to “petroleum/oil AND water AND spill” by the Scopus database (scopus.com) from 1968 to 2023.

As can be observed in Figure 1, the number of research papers related to PDP spills in water has increased significantly in recent years according to the Scopus database. This increase has resulted in an average of 950 documents published in the last two years. It is important to note that 1177 documents were published in 2005, the year of Hurricane Katrina, when, according to Coast Guard estimates, more than 5 million gallons of PDPs were spilled in the Gulf of Mexico and near-shore areas. This demonstrates the compromise of research with this key issue for society.

While considerable research exists on the detection, characterization, and containment of spills of petroleum products, there is a notable absence of in-depth studies regarding recent advancements and the significant benefits they add. Consequently, the objective of this review is to provide visibility on detection and characterization methods that are rapid and precise, along with those that employ green chemistry-based strategies. This review provides a comprehensive overview, laying the foundation for the development of novel detection and containment strategies. It fosters rapid, precise, and eco-friendly approaches to address spill-induced pollution, thereby contributing to the advancement of the field. Furthermore, it serves as a catalyst for future research endeavors. The review commences with an overview of the most classical detection approaches, including observation, and then progresses to discuss cutting-edge approaches, such as the use of sensor-based technology coupled with artificial intelligence.

Furthermore, an evaluation of the most commonly used containment and/or disposal methods was conducted, encompassing both traditional approaches, such as the utilization of skimmers, and cutting-edge strategies that incorporate the use of foams and related materials. By enhancing our comprehension of the underlying causes and pathways of contamination, as well as the detection, characterization, and mitigation strategies associated with such incidents, we can establish more resilient prevention, response, and remediation frameworks, ultimately contributing to the protection of aquatic ecosystems.

## 2. Identification and Characterization: Traditional Techniques

**Figure 2:**
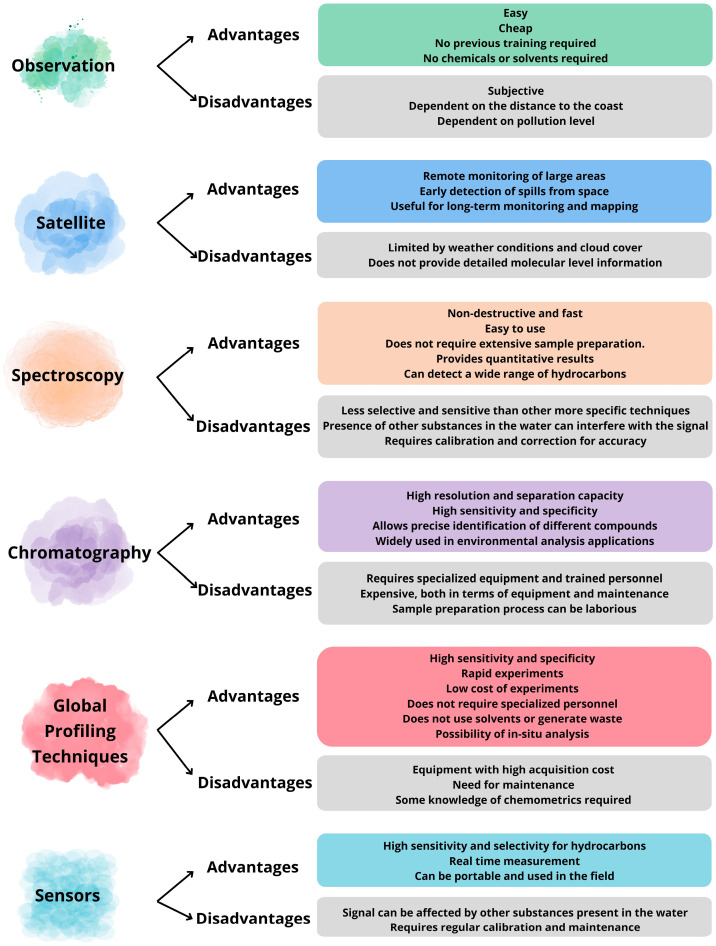
Advantages and disadvantages of classical and novel methods of detection for PDP spills.

Classical techniques have been used for a long time to detect and characterize PDP spills. An in-depth analysis of the application of these techniques in the detection of PDPs in water, focusing on their advantages and disadvantages, is summarized in Figure 2. Initially, control technicians were positioned in elevated positions in the coastal zone for shore observation. However, this method was limited to a certain distance and level of contamination and was entirely subjective. Subsequently, remote sensing techniques, such as satellite imagery and aerial surveys, were incorporated to offer broader spatial coverage and rapid detection capabilities [15,16,17]. However, challenges such as cloud cover and water turbidity can hinder the effectiveness of these techniques, necessitating the use of additional methods for reliable characterization of a spill [18,19].

Fluorescence spectroscopy has emerged as a valuable tool for the rapid and non-destructive detection of PDPs in water samples [20]. This technique exploits the characteristic fluorescence signatures of hydrocarbons, enabling sensitive and selective detection. Infrared spectroscopy also provides additional information about the chemical bonds and functional groups present in spilled substances, aiding in their identification and characterization. Indeed, both techniques have been incorporated into official methods by the Environmental Protection Agency and the American Society for Testing and Materials for the detection and characterization of PDPs in water [21,22,23]. In this context, Clark et al. [24] evaluated the use of near-infrared (NIR) spectroscopy for the detection of spilled PDPs instead of using sensing techniques, with a 30% increase in the success of detection. Wang et al. [25] also successfully applied NIR techniques to detect PDP spills from petrol, diesel, and lubricating oil in maritime waters. Baszanowska and Otremba [26] employed a fluorescence indicator based on fluorescence spectroscopy to detect PDPs in seawater within the ratio range of 50·10^−9^ to 200·10^−9^ L. Zhang et al. [27] also demonstrated the significance of laser-induced fluorescence for the detection of oil spills on the sea surface. Burlakovs et al. [28] demonstrated the efficacy of portable X-ray fluorescence spectrometry in the detection of metal ions from different pollutants that could be found in aqueous media.

Despite the favorable outcomes achieved, the utilization of these spectroscopic techniques for the detection of PDPs spilled in water, based on the identification of individual compounds or the interconnectivity of functional groups, requires a high degree of sample preparation and the possession of expert knowledge on the part of the analyst. This is due to the fact that the correct analysis of spectra requires a high degree of subjectivity and is time-consuming.

Chromatographic techniques have emerged as a key player in the detection and characterization of PDP spills. Gas chromatography (GC) [29,30] and high-performance liquid chromatography (HPLC) [31] have been used extensively for this purpose due to their unparalleled ability to analyze the composition of oil mixtures with high sensitivity and resolution. GC coupled to mass spectrometry (MS) has proven effective in identifying individual compounds in complex mixtures, while HPLC provides precise quantification and analyte separation. Adhikari et al. [32] developed a method based on GC-QToF-MS for the detection of petroleum hydrocarbons in weathered petroleum residues with important results in the detection of even traces of alkyl-PAHs. Similarly, Radovic et al. [33] employed GC-MS/MS for the detection of PDP spill residues in the Gulf of Mexico with promising results. Stanford et al. [34] also utilized MS with Fourier transform for the analysis of spilled PDPs, discovering that the most prevalent water-soluble compounds are acidic oxygen-containing compounds and basic nitrogen-containing compounds. In addition, Yang et al. [35] employed HPLC to assess the weathering process in spilled PDPs, demonstrating the persistence of the naphthenic acid fraction per the number of carbons and oxygens atoms. Nemirovskaya et al. [36] demonstrated the suitability of the HPLC technique for the detection of organic compounds in sediments in water exposed to PDP spills, characterizing the nature of the PDPs.

As previously discussed, the analytical techniques utilized for the detection and characterization of PDP spills in water have been successfully employed for years. However, these techniques come also with significant disadvantages. For instance, techniques based on image recognition are highly conditioned by weather conditions or water surface turbidity. Conversely, spectroscopic and chromatographic methods require specialized instrumentation, particularly for sample preparation, limiting their in situ field applicability due to logistical constraints like the need for vacuum pumps and specific sample preparation protocols prior to analysis. Furthermore, conclusions are based on the identification of individual compounds in each product, which entails time-consuming and costly experiments. Conversely, this type of data processing, which involves the identification of individual compounds, has proven to necessitate a significant degree of experience and training on the part of the analyst and to be highly subjective on numerous occasions.

Overall, while traditional techniques have been historically instrumental in the detection and characterization of PDP spills in water, providing invaluable information on the nature and extent of environmental contamination, there is a growing need for more objective approaches that are user-friendly and suitable for in situ applications. This has prompted researchers to intensify their endeavors to achieve such objectives.

## 3. Identification and Characterization: Sensor Approaches

In recent years, technological progress has played a key role in the issue of water pollution by petroleum products with important advantages (Figure 2). However, the integration of new statistical tools, such as complex algorithms or the use of machine learning tools, represents a notable advance in research related to the detection and characterization of PDPs spilled in water [37].

In this way, the approach of general profile techniques arose, through which researchers employed techniques with a wide sensitivity and selectivity for the detection and characterization of PDPs spilled in water. These techniques included chromatographic (GC or HPLC), spectrometric (MS), or spectroscopic (fluorescence and infrared) techniques. Instead of seeking individual characterization, the focus was on all the signals at the same time. For instance, in the case of the MS, each *m*/*z* ratio served as a sensor whose signal corresponded to the measurement collected by said sensor. Consequently, a total spectrum within an *m*/*z* range corresponded to a set of sensors collecting signals at the same time. In this way, approaches such as the Total Ion Chromatogram (TIC) or the Total Ion Mass Spectrum (TIMS) began to be used [38,39]. These approaches, when combined with chemometric tools, offer enhanced sensitivity, specificity, and efficiency, enabling rapid and accurate assessment of spill events and their environmental impacts [40]. A summary of the most recent research with sensor-based approaches can be found in Table 1. 

In brief, Ferreiro et al. [41] developed a method using headspace MS (HS-MS) and the TIMS approach to detect and characterize spilled petroleum-derived products. Chemometric tools were employed to develop characteristic fingerprints for each of the PDPs used, resulting in a 100% successful characterization in minutes. Wang et al. [42] employed GC-MS to detect the presence of PDPs in a real case study of the Dalian petroleum spill incident in China. In this instance, they applied a range of chemometric tools, including Hierarchical Cluster Analysis (HCA) and Principal Component Analysis (PCA). Jaén-González et al. [43] developed a method based on headspace ion mobility spectrometry (HS-IMS) using the general profile approximation. In this method, each drift time of the ion mobility corresponds to a sensor, and the intensity obtained corresponds to the ion mobility signal. This approach, combined with chemometric tools, enabled the detection and characterization of PDPs at a minimum concentration of 4 ppb, which is well below the limit established by the Ministry of Spain for water to be considered contaminated (i.e., 8 ppb). Mitkidou et al. [44] employed GC from a TIC approach combined with chemometric tools for the detection and characterization of PDPs spilled in water and sediments of the Nestos River Estuary in Northern Greece. This approach enabled the discrimination of five PDPs, namely jet fuel, gasoline, diesel, heating oil, and bunker fuel.

Spectroscopic techniques have also been employed in these combined approaches. For instance, De Kerf et al. [45] demonstrated the suitability of thermal infrared images combined with machine learning for the detection of PDP spills with 89% accuracy. Mirnaghi et al. [46] used fluorescence spectroscopy and a chemometric tool (PCA) to successfully characterize 130 PDPs spilled in water. This model was validated using traditional GC-MS analysis.

In conclusion, the integration of sensors and chemometrics offers a synergistic approach to spill monitoring and management, providing a comprehensive toolkit for environmental scientists and regulatory authorities. Real-time sensor data can be processed and analyzed in situ or remotely using advanced chemometric models, enabling timely decision-making and adaptive response strategies. Furthermore, the development of sensor networks and autonomous monitoring systems enables continuous monitoring of vulnerable water bodies, thereby improving early warning capabilities and reducing the ecological impacts of spills. It can be therefore anticipated that recent advances in the implementation of complex algorithms, such as machine learning, in techniques with great technical advantages, such as the use of sensors or the approach using general profiles, represent the future of research for the detection and characterization of spilled PDPs. In this sense, it is important to highlight that both the sensors and the general profile approaches eliminate the disadvantages previously associated with traditional techniques, allowing faster, inexpensive, and simple procedures that do not require highly specialized personnel. Moreover, a significant proportion of these techniques operate at atmospheric pressure, which enables in situ analysis or, as previously mentioned, real-time characterization.

## 4. Removing/Containing Actions

Once the compounds have been identified and quantified, the next step is to focus on their removal, with particular attention to PDPs. Due to their persistence and recalcitrant nature, PDPs are priority pollutants to be removed from ecosystems [47]. Nowadays, the array of available oil spill cleanup technologies can be classified into four principal categories: chemical remediation, which encompasses dispersants and emulsion breakers; thermal remediation, which involves in situ burning; physical/mechanical remediation, which utilizes booms, skimmers, and adsorbents; and bioremediation, which makes use of naturally occurring microorganisms to decompose PDPs. In order to achieve the most environmentally favorable and cost-effective response, it is recommended that an integrated remediation approach be considered [48,49]. 

The following section will review the most common (classical) methods for the removal, containment, and degradation of PDPs. It will then proceed to examine novel strategies, with a particular focus on bio-based systems. In addition, a more in-depth study of the advantages and disadvantages of the classical and novel methods is included (Table 2).

### 4.1. Classical Methods for Removal of PDPs

Despite a few minor improvements, classical methods for the removal of PDPs from water media remain virtually unchanged. Nonetheless, the most classical methods, such as booms and skimmers, have proven their efficiency and continue to be used worldwide. In general, oil is less dense than water and will spread over its surface. To limit the oil spreading and contain it, stationary floating devices designated as booms are applied. These are physical barriers that confine the oil by maintaining an adequate thickness, which is necessary to implement further remediation actions. When deployed at sea, booms are anchored and towed into the spill area in a configuration that allows them to act as a physical and floating mechanical barrier. This configuration is formed by the current and waves, and it pushes the oil toward the center of the boom. The main goal of the booms is to contain oil slicks at sea, thus limiting their spreading, evaporation, fragmentation, dissolution, natural dispersion, and emulsification. As a result of the oil being redirected to a location where it can be recovered, collected, and treated, biologically sensitive areas can be better protected [47,49,50,51]. The selection of the most suitable technique to apply is dependent on the location of the spill. Indeed, fireproof booms have been developed to contain oil spills when in situ burning is considered a suitable option. While they are not commonly employed in the marine environment, they are considered the primary spill response procedure in icy waters, namely in the Arctic. A large-scale field experiment and several mesoscale oil burns conducted by Fritt-Rasmussen et al. demonstrated that the burn residue found at the burn sites was markedly reduced after four days in seaweed vegetation. This was attributed to the concentration of oil compounds being found below ecotoxic concern levels [52,53]. However, the combustion of the oil results in high volumes of toxic gaseous emissions, and, depending on the size of the spill, the procedure can take several days with several simultaneous fires. Given this, monitoring emissions and assessing the health and environmental impacts becomes compulsory [54,55]. While possessing some drawbacks, these traditional methods can be further improved by developing cost-effective, efficient, and environmentally friendly oil–water separation technologies (Table 2). 

Skimmers are mechanical devices designed to remove floating oil from water. They are used in conjunction with booms and are available in a variety of forms, including belts, discs, mops, drums, and even floating suction set-ups. Depending on the characteristics of the area and the operation principle, skimmers may be self-propelled, dynamic, or stationary. In general, these devices are classified as weir, oleophilic, and suction skimmers [47,48,49]. Weir skimmers function in a manner analogous to a dam, collecting the floating oil through the action of gravity or a pump. These systems are more effective with less viscous and lower-density oils, although they are susceptible to jamming and coggling by floating debris. Oleophilic skimmers are constructed from materials with oleophilic properties, which can be shaped like disks, brushes, or belts. The oil will then adhere to the surface of the material, from which it can be removed and subsequently stored. While not prepared to deal with mixtures of oil and dispersants, flexible oleophilic skimmers can operate on rough ice and debris and maintain their efficiency regardless of the oil thickness and are less influenced by waves. Suction skimmers are vacuum pump systems with an air venture for conducting the oil through wide floating heads and transferring it to storage tanks. Despite a few technical limitations that can be caused by debris, and the fact that they are not advisable for use with inflammable oil products, suction skimmers are quite efficient in handling a wide range of oil viscosities. Overall, the effectiveness of a skimmer is determined by how fast and well the oil is removed, as well as the amount of water collected in the mixture [48,51]. Recently, Abidli et al. [56] developed a pilot-scale skimmer prototype for continuous and simultaneous oil–water separation, regeneration of the used sorbent materials, and selective removal of oils and organic solvents from oil–water mixtures. The system combines a hydrophobic–oleophilic porous sorbent material-based separation bed and a vacuum-assisted separation unit. It is claimed that this novel apparatus will be able to provide easy, reliable, and affordable operation with minimal maintenance. Furthermore, the developed design can also be expanded to remove organic and inorganic contaminants from wastewater through absorption and/or desorption processes.

The initial hours following an oil spill represent a pivotal period during which the efficacy of oil recovery, removal, and disposal of the oil, as well as the associated environmental impact, can be effectively gauged. Ideally, the initial response should aim to halt the spread, evaporation, and emulsification of the oil. The most common initial actions rely on the deployment of booms, which cannot prevent the evaporation of the more volatile hydrocarbons or the oil emulsification. However, the formation of small oil/water droplets hinders the cleaning process and oil recovery. After a skimming operation, adsorbent materials are usually applied to facilitate oil removal, comprising natural organic, inorganic, and synthetic materials [51,57,58]. In search of a practical and efficient oil recovery method, Nam et al. [59] developed an absorbent from a mixture of two polyolefin polymers, a low-density polyethylene and an elastomer. The system presents a fast absorbing kinetics capable of stopping the crude oil weathering process in open waters. After absorption, the crude oil and the absorbent form a gel, which the authors propose removing using a skimmer. Moreover, the formed gels exhibit a low water content, which allows the recovered crude oil to be refined through standard procedures rather than being disposed of as chemical waste. Apart from organic compounds, inorganic sorbents present a wide range of materials divided into four main groups, according to their chemical composition. Silica sorbents include volcanic glasses, gaizes, diatomite, and perlite; the carbonaceous sorbents group comprises carbon nanotubes, silica–carbon materials, activated carbon, hard coal, and graphite; carbonate sorbents consist of limestone, pyrocarbonate, and carbonate rocks; and aluminosilicate sorbents are represented by ash, glauconite, zeolite, vermiculite, and aluminosilicate [60]. Similar to organic adsorbents, mineral adsorbents are cost-effective and abundantly available, with the capability to absorb between 4 and 20 times their weight in PDPs [61]. Schrader et al. [62] conducted an assessment of the adsorption capabilities of mineral, organic, and synthetic adsorbents. Their findings revealed that synthetic adsorbents exhibited the highest adsorption capacity, while inorganic adsorbents also demonstrated notable adsorption efficiency. Moreover, Hoaghia et al. [63] demonstrated the efficacy of zeolite in removing PDPs (persistent dissolved pollutants) from water, highlighting that zeolites with smaller particle sizes (<10 µm) exhibit superior adsorption capacities compared to those with larger granule sizes (1–3 mm). Magnetite nanoparticles and magnetite silica nanocomposites were applied by Zabermawi, Nidal M. et al. in the treatment of oil-refinery-contaminated wastewater. Despite a very high initial pollutant concentration, the procedure generated high-quality effluents in only 2 h. This work demonstrated the efficacy of mineral-based nanoparticles and composites when applied for the removal of PDPs from contaminated water, showing how these can be a promising and economic method for the treatment of highly toxic and complex industrial wastewater such as petroleum refinery effluents [64]. Adsorbents based on activated carbon have been recently researched by Jumroonrat et al., providing insights into the recovery of green solvents contaminated with PDPs. Batch experiments demonstrated that granular activated carbon can be applied in an industrial setting for the recovery of solvents in a manner that is more environmentally friendly than traditional methods of oil contamination management. Although the authors did not consider the appropriate disposal method for spent adsorbent and the recovery of adsorbed oil for reuse, they proposed that granular activated carbon could be used to reduce the operational costs of the drilling procedure [65].

Another alternative strategy for oil disposal is the application of chemical dispersants. These are typically composed of surfactants and a solvent and are becoming an increasingly accepted approach due to their suitable and efficient response in deep water or adverse weather conditions. After an oil slick is sprayed with a dispersant, the interfacial tension between the oil and water is reduced, and finely dispersed oil droplets are formed. This process makes oil less likely to stick to surfaces, increasing the surface area of the spill droplets and accelerating their rate of natural biodegradation [48,51,66]. However, dispersants also present some drawbacks, as they can be toxic due to the use of petroleum-based surfactants, such as dioctyl sodium sulfosuccinate salt, and hydrocarbon-based solvents, such as 2-butoxyethanol. To overcome this, the use of greener surfactants is required. For example, Nyankson et al. [67] tested the effectiveness of food-grade surfactants, including Span 80, Tween 80, and modified soybean lecithin, on a dispersant formulation, together with naturally occurring halloysite nanotubes. The prepared dispersant demonstrated a 99 vol % dispersion effectiveness, indicating the high dispersion effectiveness of an environmentally friendly dispersant. The evaluation of the toxicity of crude oils and dispersants on the aquatic environment, specifically their phytotoxic concentrations, constitutes a reactive response to large oil spills in marine waters. However, the majority of this information has been mainly obtained from post-oil-spill investigations, while the toxicity correlations between most aquatic plants and fauna species remain largely unknown. As the majority of the reported phytotoxic data for dispersed oils and dispersants pertain to saltwater plants and dispersants that are no longer in use, this data gap must be filled. Ideally, this would be achieved by testing sensitive species of algae, plants, and marine animals and microorganisms to determine threshold concentrations. The greater the data availability is, the more consistent and relevant the database can be in assisting a larger-scale evaluation for managing and sustainably maintaining aquatic ecosystems [68,69,70,71,72].

Fortunately, these ecosystems are also inhabited by naturally occurring strains of bacteria, fungi, and archaea that can degrade PDPs. As previously mentioned, the action of a dispersant usually facilitates the microorganisms’ action and consequently the oil biodegradation by reducing the oil droplet size and increasing its surface area. Most of the PDPs can be used as a carbon source by microorganisms. From alkanes to high-molecular-weight polycyclic aromatic hydrocarbons, consortiums of native bacterial species act together to break down the oil complex hydrocarbon mixture, converting it into carbon dioxide, water, and inert residues [48,66]. Nevertheless, biodegradation of PDPs from an oil spill is a slow process, with a lag phase reaching 2 to 4 weeks and taking months or years to be completed even after biostimulation. Consequently, an efficacious bioremediation process necessitates an initial screening of the PDP-degrading microorganisms present at the spill site, with the addition of fertilizers also being employed to ensure the requisite nitrogen and phosphorus requirements for their growth. The first fertilizers were added to enhance the bioremediation process following the *Exxon Valdez* oil spill, with the aim of assisting in shoreline cleaning. Between 1989 and 1991, approximately 48 tons of nitrogen and 5 tons of phosphorous were applied, representing 2237 separate shoreline applications of fertilizer. The mass of residual surface oil decreased by approximately 28% per year, while sub-surface oil decreased by 12% per year [47,48,51,73]. However, further laboratory-scale tests are required to gain a better understanding of how factors such as sediment displacement, oxygenation, and droplet size influence the bioremediation kinetics over time. Prince et al. demonstrated that the biodegradation of dispersed oil is accelerated when the oil is present at the optimal concentration for the successful application of dispersants [74]. Conversely, Brakstad et al. developed a laboratory system to investigate the biodegradation of small oil droplet dispersions (10 μm to 30 μm), highlighting the significance of oil droplet size for biodegradation [75].

**Figure 3:**
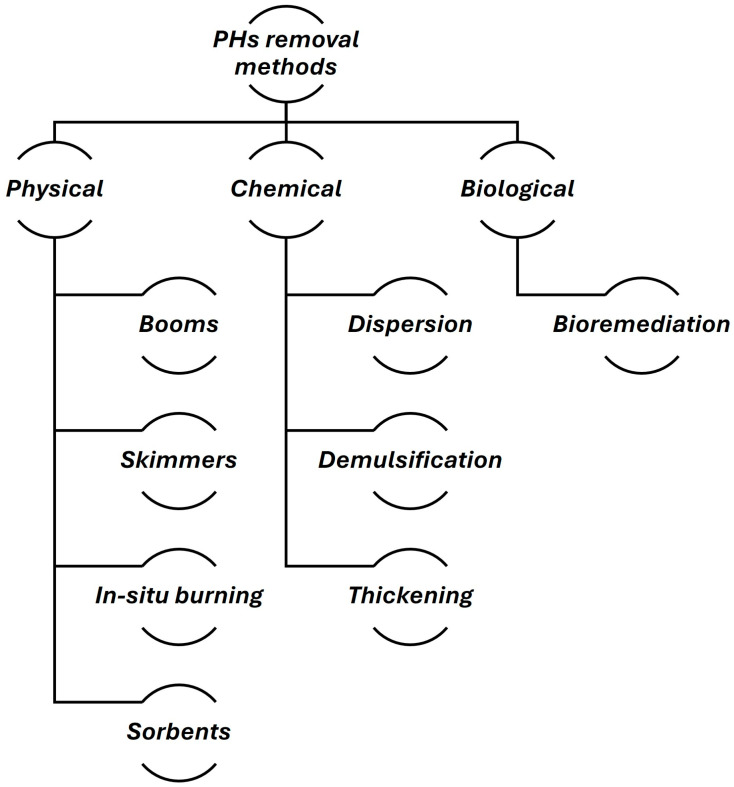
Classical methods for oil spill response and removal of PDPs.

In conclusion, the classical methods of oil spill response (Figure 3) remain the foundational approach, as ongoing research and innovation are essential to develop more efficient, environmentally friendly, and sustainable solutions to mitigate the impacts of oil pollution on aquatic ecosystems.

### 4.2. Bio-Based Materials in Oil Spill Remediation

The utilization of scientific knowledge developed in various fields has led to significant advancements in the field of oil spill response technologies through the incorporation of bio-based materials. In particular, natural polymers have gained increased interest as functional materials for oil–water separation due to their natural abundance, biocompatibility, sustainability, and the presence of a large number of hydrophilic functional groups. Different biopolymers, such as polysaccharides, are suitable candidates due to their highly favorable properties [76]. A prominent example is chitosan, which is obtained from the deacetylation and depolymerization of chitin. The amino and carboxyl groups found in chitosan render it an effective biosorbent for water pollutants, including pharmaceuticals, toxic metals, dyes, microplastics, and oil compounds. Although chitosan is well-known as an adsorbent, it has been modified to enhance its pollutant removal capacity, with a particular focus on improving its mechanical, antimicrobial, and thermal properties [77,78,79,80]. Chitosan has been used as beads, aerogels, foams, and membranes, and further chemically modified by carboxylation, alkylation, or quaternization reactions, among others [81,82]. Concerning oil–water separation, chitosan has been extensively employed due to its capacity to enhance the wetting properties of porous media. Superoleophilic and oleophobic or superhydrophilic and hydrophobic chitosan-based materials serve as a good example, demonstrating the potential of sustainable and eco-friendly alternatives for oil–water separation methods. In a similar vein, Meng Wang et al. prepared an underwater superoleophilic cotton fabric doped with chitosan, which demonstrated remarkable oil–water separation capacity. This material could be recycled up to seven times while retaining its high performance even under harsh conditions [83]. Chitosan-based aerogels designed by Qiaozhi Wang et al. [84] were found to be suitable for separating oil–water mixtures while simultaneously removing copper ions from water. The aerogels were produced by freeze-drying chitosan/genipin gels containing chitosan-coated microbubbles, generated by applying high-intensity ultrasounds. The systems also exhibited superhydrophilicity/underwater oleophilicity, as well as anti-fouling capacity. Furthermore, chitosan was employed as an additive in composite paper by Ling et al. [85], facilitating the fabrication of a highly durable, superhydrophobic separation material with additional antimicrobial properties. Chitosan and cellulose have been extensively utilized in the fabrication of aerogels and membranes capable of separating oil–water mixtures, which are commonly prepared through simple methodologies, resulting in low-cost and environmentally friendly bio-based materials [86,87,88,89] (Table 2). 

Another biopolymer worthy of mention is cellulose, which has attracted considerable attention for its potential use in oil–water separation purposes. This is the most abundant and renewable biopolymer with a vast range of applications [90]. Cellulose-based superhydrophilic materials are typically composed of polymers, such as polysulfone (PSF), polyacrylic acid, or polyvinylpyrrolidone (PVP), though these are prone to contamination, lack mechanical strength, and are difficult to modify. Inorganic metallic materials, such as alloys, metals, and ceramics, have also been mixed with cellulose, but they typically exhibit low separation efficiency. Notwithstanding these considerations, the structural characteristics of cellulose-based materials facilitate the efficient separation of oil/water mixtures, while simultaneously preventing the generation of secondary pollution [91,92]. A produced membrane exhibited superhydrophilic/underwater superoleophobic properties and was capable of efficiently separating surfactant-stabilized oil-in-water emulsions, as well as rapidly removing dyes from water [93]. Magnetic hydrophobic cellulose-modified polyurethane filter foams were prepared by Alazab et al., showing great oil–water separation properties, even in complex environments, such as acidic media and simulated seawater. The produced materials exhibited a fast diffusion of oil while preventing water diffusion through them during the separation process. Furthermore, the authors demonstrated that the magnetic and hydrophobic properties, as well as the oil absorption capacity, were enhanced by the addition of iron (II, III) oxide nanoparticles to the modified cellulose–polyurethane foams [94]. Nevertheless, polymer-crosslinked membranes continue to present several challenges, namely membrane fouling, high cost of production, elaborate processing, and low degradability. Overcoming such issues will contribute to the development of more attractive materials, as has been demonstrated by Li et al. in their preparation of all-natural self-crosslinking cellulose membranes [95]. A straightforward method, based on physical interactions and entanglements among the extracted cellulose fibers, enabled the authors to create biodegradable, sustainable, low-cost, and reusable superhydrophilic/underwater superoleophilic cellulose membranes. 

Cellulose-based materials have also been modified by the addition of microorganisms that are able to degrade PDPs. This approach addresses the challenge of efficient PDP elimination by combining bio-based adsorbents with bioremediation, via loading materials with, for instance, bacteria capable of degrading PDPs. In this context, Zapata et al. have developed carbon microspheres with immobilized *P. stutzeri* cells for the biocatalysis of PDPs and crude oil from oil–water emulsions as an alternative method to the conventionally used processes in the oil and gas industries. It was observed that the removal of a mixture comprising benzene, toluene, and phenol was the highest for the support material without *P. stutzeri* (100%), followed by the biomaterials with bacterial cells (84.8%), and the lowest efficiency was shown for the free *P. stutzeri*. When removing crude oil, both the support and the biomaterials led to the complete removal of the PDPs from oil–water emulsions. Bacterial catalysis was confirmed by the presence of light hydrocarbons, which resulted in the bioconversion of the tested PDPs into more oxidized and less toxic products [96]. Similarly, sponges made from nanocellulose were loaded with microorganisms by Xu et al., and this proved to be an effective method for the efficient removal of PDPs from model polluted water [97]. The prepared microporous sponges demonstrated the capacity to provide sufficient space and nutrients for the growth of microorganisms, displaying a synergistic effect when mixed strains are present. These systems are capable of degrading approximately 95% of the simulated PDPs during the initial 96 h. 

In addition to being synthesized by plants, cellulose can also be obtained through fermentation by certain bacteria, such as *Acetobacter xylinum*. In comparison with plant cellulose, bacterial cellulose (BC) is typically characterized by high crystallinity, a high degree of polymerization, high purity due to the absence of lignin or hemicelluloses, and superior mechanical properties. However, its application is limited when used as an adsorbent or oil–water separation material due to the densely packed mesh of nanofibrils, which can hinder solvent infiltration and diffusion. To enhance the separation performance of BC, numerous efforts have been made by changing BC incubation conditions, blending, crosslinking, dip-coating, or composite preparation. Galdino et al. developed BC membranes for the treatment of oily waters using an alternative medium containing 2.5% corn steep and demonstrated how fermentation time influenced the membranes’ mechanical strength and filtration flow rate [98]. Superhydrophilic/underwater superoleophilic BC membranes have been prepared by Wahid et al. by blending BC nanofibers with silica nanoparticles, further modified by a polydopamine coating. These membranes exhibited a high oil–water separation efficiency in a high flux with small negative pressure, as well as anti-fouling properties, the ability to be recycled, and superior stability under harsh conditions [99]. Hu et al. demonstrated the remarkable efficiency of highly stable ultrafiltration membranes through the modification of the BC 3D network by polymerizing dopamine with purified BC sheets and forming composites through the integration of graphene oxide [100]. Despite the documented high performance of the aforementioned materials, Li et al. took the binding capacity of cellulose to water molecules into consideration in order to improve BC crystallinity while increasing the water flux. To this end, the purified BC hydrogels were soaked in a solution of cellulose nanocrystals, resulting in the formation of new hydrogels with improved oil–water separation capacity. This enhancement was attributed to the decrease in accessibility of the hydrogels to water molecules due to the increase in the crystallinity degree [101]. As previously demonstrated, BC-based materials prepared for oil–water separation have numerous common features, including high separation efficiency, anti-fouling properties, recyclability, and biodegradability. These promising candidates therefore warrant further investigation, to achieve reliable scale-up and applications in real-life scenarios [102,103].

In addition to the synthesis of BC from agro-industrial waste, biomass-derived materials and residues also have the potential to be directly applied in the removal of PDPs. The use of agro-industrial waste has been recently explored by Valdivia-Rivera et al. for bioencapsulation and bioremediation purposes. Proteins were isolated from mango and red bean and were further used in the preparation of macrocapsules. Subsequently, the consortium of microorganisms was encapsulated with the aforementioned macrocapsules. The bioremediation assays, conducted using hexadecane and diesel, as model pollutants, demonstrated a reduction in the time required for hydrocarbon uptake when the bacterial consortium was encapsulated. The consortium was able to remove 90 to 100% of the tested PDPs [104]. Other biomass options can include the use of biochar, as demonstrated by Silavani et al. through a comparison with activated carbon and the characterizing of biomass-derived materials for the adsorption of toluene from water. The results indicated that the biochar material could be considered a green and cost-effective option, as it demonstrated faster removal kinetics for toluene, which was highly sorbed onto the biochar surface [105]. A recent study demonstrated the efficacy of water hyacinth biomass as a viable sorbent for the removal of PDPs. The biomass was mixed with varying quantities of coconut oil to increase its hydrophobicity and was then capable of removing above 90% of a simulated oil spill, even after a third sorption–desorption cycle [106].

In addition to all the aforementioned methods, photo-dissolution can transform insoluble components from crude oil at sea into water-soluble products. However, the reactions initiated by sunlight, which could have opposing effects on different groups of oil compounds, are not well understood. Nevertheless, oxidation reactions may occur, contributing to the degradation of PDPs. Freeman et al. evaluated the importance of photo-dissolution on the fate of Macondo oil (the oil spilled from the *Deepwater Horizon*), demonstrating that photo-dissolution has a significant impact, comparable to other widely recognized fates [107]. After exposing oil from a natural seep and spill oil from the Refugio spill to simulated sunlight, Snyder et al. demonstrated that exposure to sunlight radiation facilitates the dissolution of surface oil. Although the verified effect was more pronounced on the seep oil, even after 67 days of exposure, 488 persistent water-soluble compounds remained in the spilled oil, representing a prevalence that was more than three times greater than that observed in the seep oil. These findings also highlighted the importance of future work for verifying the toxicity, carcinogenic effects, and ecosystem health impact of the identified compounds [108]. 

Furthermore, the combination of photochemistry-based approaches with other response methods could result in a positive synergistic effect. For instance, the application of simulated sunlight radiation to a chitosan-based aerogel loaded with cuttlefish ink particles demonstrated the improved efficiency of sunlight in conjunction with an adsorbent, as reported by Guan et al. A sunlight intensity of 1 kWm^−2^ was sufficient to elevate the temperature of chitosan-based aerogels up to 70 °C within 60 s. This enables the aerogel to absorb 18 times its weight in crude oil, which can then be recovered via extrusion or in conjunction with a vacuum pump device. Furthermore, the aerogel biodegrades within 30 days without causing environmental pollution [109]. Another potential application was examined by Gbogbo et al. [110] when fabricating a halloysite nanotubes composite containing a binary surfactant mixture of lecithin and Tween 80, which was then exposed to UV light irradiation when in contact with crude oil. The results demonstrated that the composite was capable of photodegradation of the crude oil aromatics and asphaltene fractions, accompanied by the formation of intermediate photodegradation products, in accordance with the previously reported findings. Despite the current lack of knowledge regarding the formation of intermediate products and photodegradation pathways, photodegradation represents a promising remediation method that could be readily integrated with other approaches, potentially enhancing their efficacy.

The integration of bio-based materials, microorganisms, and photochemical processes may present a multifaceted strategy toward more effective and sustainable oil spill remediation [111]. Continued research and development in this area may offer a promising avenue for addressing environmental challenges posed by oil spills while promoting eco-friendly solutions for mitigation and cleanup.

It is also important to note that while the primary objective of cleanup and containment strategies is to remove as many of these contaminants as possible from the marine environment and reduce their potential environmental impact, one of the current areas of significant research regards the reuse of both containment pathways and petroleum products. In this context, while skimmers have demonstrated significant potential, the methods used for the separation of PDPs and their subsequent treatment for reuse are costly, time-consuming, and energy-intensive. However, more recent bio-based sorbents and gels with simpler structures, as well as adaptability to the nature of the PDP, facilitate such separation and reuse of both the sorbents and the separated PDP by reintroducing them back into the refining process [108]. It can be anticipated that another advantage of this remediation strategy is that it favors the circular economy. This is evidenced by the reduction in contamination of the spill area and the generation of new PDPs or containment products, as these materials are not discarded as waste.

## 5. Conclusions and Future Perspectives

The exponential development of petroleum-derived products, coupled with their incorporation into a diverse range of materials, has led to the blending of different products and the creation of increasingly complex compositions. This represents a significant challenge for the detection and characterization of waterborne spills, particularly in time-sensitive scenarios where the potential environmental impact of these products on water bodies is substantial, leading to dramatic repercussions for aquatic fauna and flora and human health. Conventional techniques reliant on the identification of individual compounds suffer from inherent limitations, including time-intensive and subjective methodologies, as well as the need for elaborate and costly sample preparations, occasionally involving solvent use.

The necessity for novel approaches regarding the detection of spilled petroleum products in water which are more expedient, cost-effective, and environmentally conscious than classical methods is paramount in addressing contemporary environmental challenges. In this context, pivotal analytical techniques based on global profiles, which avoid the need for individual compound identification and simulate the behavior of the sensors, are presented as highly promising alternatives. The speed and sensitivity of these new technologies permit the timely detection of contaminants, which in turn facilitates more agile and accurate decision-making in emergency situations. Reducing the time needed to identify and quantify oil spills in water is crucial for minimizing the spread of contamination and its environmental impacts. Furthermore, as these methods are more economical and environmentally friendly, their widespread use in the continuous monitoring of water bodies is encouraged, thus contributing to more effective and sustainable management of our water resources.

In another hand, despite minor advances, classical methods continue to be widely used as a remediation response for the removal of PDPs from water. However, these have proven their efficiency in containing and removing oil slicks from surface water. Nevertheless, there is still room for improvement. The development of more cost-effective, efficient, and environmentally friendly oil–water separation technologies could be used to increase the effectiveness of traditional technologies, such as dispersants, booms, and skimmers, while minimizing their drawbacks. Innovative approaches focused on transforming and applying bio-based materials, such as chitosan and cellulose from plants and bacteria, among others, show much promise given their oil–water separation efficiency, biodegradability, and sustainability profiles. However, there is still much to be understood in terms of photodegradation pathways, identification, and characterization of the originated products as their environmental and health impacts are not yet fully understood. Similarly to many of the currently large-scale applied responses, the environmental impact of experimentally proven remediation technologies should also be addressed by studying the toxicity of new dispersants, the degradation pathways of oil components, and the ecological consequences of the different remediation techniques. Combining multiple responses, such as adsorption/bioremediation or adsorption/photo-dissolution, could result in synergistic effects with the capacity to enhance the overall efficiency of an oil spill cleanup action. Research in this field should investigate the potential for synergistic effects between different remediation techniques and optimize their integration for maximum effectiveness in order to provide an adequate and faster response. This comprehensive review has also demonstrated that there is a current need to scale up innovative technologies, such as bio-based dispersants, adsorbents, and absorbents, as photo-dissolution techniques, given their relatively simple preparation and application and minimal environmental impact when compared with older methods.

## Figures and Tables

**Table 1:** Current research in the use of analytical techniques with approximation as sensors.

Authors/Reference	Samples	Analytical Technique	Statistical Tool	Results
Ferreiro et al. [41]	70 PDPs: aromatic products, alcohol, normal alkanes, gasoline, diesel, lubricants, and paraffinic products.	HS-MS	TIMS, HCA, and LDA	100% discrimination
Wang et al. [42]	Six refined petroleum products from ships. Six samples Jinshatan Beach and Haibei Square.	GC-MS	HCA and PCA	82.77% discrimination between samples
Jaén-González et al. [43]	16 PDPs in water: gasoline, diesel, lubricant and kerosene. Concentrations 8, 4, and 2 ppb.	HS-IMS	HCA, PCA, and LDA	100% identification and characterization at 8 and 4 ppb. 100% identification at 2 ppb
Mitkidou et al. [44]	Five petroleum product samples (jet fuel, gasoline, diesel fuel, heating oil, and bunker fuel) and 15 and 6 water and sediment samples from Nestos River	GC-MS	TIC	Completely difference between PDP samples. No PDPs detected in sediments and water samples
De Kerf et al. [45]	Controlled oil spill in port	IR	Convolutional neural network	89% discrimination
Mirnaghi et al. [46]	130 PDPs in different evaporative states	Fluorescence Spectroscopy	Parallel factor analysis and PCA	Complete differentiation between PDP samples. Method validated by official method GC-MS

**Table 2:** Advantages and disadvantages of classical and novel PDP removal methodologies.

Method	Advantages	Disadvantages
Skimmers and Floating Barriers	Effective for recovering large quantities of floating oil on the water surface.Relatively fast and simple to deploy. Can be used in calm waters and some weather conditions.	Limited to surface oil recovery. Less effective in turbulent or rough waters. Require maintenance and continuous monitoring.
Controlled Burning	Quick to reduce the amount of oil on the water surface. Can be used in remote or difficult-to-access areas. Does not generate additional waste beyond combustion emissions.	Can produce air pollutants and noxious gases. Not effective for large spills or in environmentally sensitive areas. Requires specific safety and control conditions.
Dispersants	Help break up large oil slicks into smaller droplets. They facilitate the natural degradation of oil by microorganisms. Can be applied by plane or ship to cover large areas.	Potentially toxic to marine life and the aquatic ecosystem. They do not physically remove the oil, only disperse it in the water. Limited effectiveness depending on spill conditions and type of oil.
Bioremediation	Uses microorganisms to degrade oil naturally. Lower environmental impact compared to chemical methods. Can be applied in shallow waters and shorelines.	Slow process, taking weeks or months to be completed. Limited effectiveness in cold or low-nutrient-concentration waters. Requires continuous monitoring to evaluate effectiveness.
Absorbents	Physically removes oil from water and shorelines. Can be used in sensitive areas where other methods are impractical. Requires simple equipment. Possibility of reuse of both absorbent and PDP.	Generates solid waste that must be disposed of properly. Costly and laborious, especially in large spills. May cause disturbance to habitat and marine life during cleanup.

## Data Availability

Data will be available under requirement.
